# Cloning and Functional Determination of Ammonium Transporter PpeAMT3;4 in Peach

**DOI:** 10.1155/2020/2147367

**Published:** 2020-12-03

**Authors:** Shuanghong You, Yuqing Wang, Yanju Li, Yuhe Li, Ping Tan, Zheng Wu, Wenjing Shi, Zhizhong Song

**Affiliations:** ^1^Chongqing Academy of Agricultural Sciences, Chongqing 401329, China; ^2^Key Laboratory of Molecular Module-Based Breeding of High Yield and Abiotic Resistant Plants in Universities of Shandong (Ludong University), Yantai 264025, China; ^3^College of Agriculture, Ludong University, Yantai 264025, China; ^4^Yantai Academy of Agricultural Sciences, Yantai 264000, China

## Abstract

Ammonium (NH_4_^+^) plays key roles in plant growth, development, fruit quality, and yield. In plants, NH_4_^+^ uptake and transport are facilitated by NH_4_^+^ transporters (AMT). However, molecular mechanisms and physiological functions of type-II AMT (AMT2) transporters in fruit trees are still unclear, especially in peach. In this study, we cloned and characterized an AMT2 family gene from peach, *PpeAMT3;4*, and determined its function in yeast mutant. Expression analysis showed that *PpeAMT3;4* was majorly expressed in peach roots and significantly decreased by NH_4_^+^ excess but had no response to NH_4_^+^ deficiency. Functional determination and ^15^nitrogen-labeled NH_4_^+^ uptake assay in yeast cells implied that PpeAMT3;4 was a typical high-affinity transporter, with a *K*_*m*_ value of 86.3 *μ*M, that can uptake external NH_4_^+^ in yeast cells. This study provides gene resources to uncover the biological function of AMT2 transporters and reveals molecular basis for NH_4_^+^ uptake and nitrogen (N) nutrition mechanisms in fruit trees.

## 1. Introduction

Ammonium (NH_4_^+^) is the preferred form of nitrogen (N) source absorbed by both annual and perennial plant species, especially in N-deficient soils [1, 2]. However, with the increasing soil N input and atmospheric deposition, plants have to deal with NH_4_^+^ stress from sources below and above the ground [1–6]. Thus, an optimal amount of NH_4_^+^ should be effectively absorbed from the soil via plant roots, to sustain basic growth demands.

Extensive studies in angiosperms [3–5] and in basal land plant liverwort [6] indicated that NH_4_^+^ acquisition and uptake were performed by NH_4_^+^ transporters (AMT) through the plasma membrane of root cells. The first plant AMT family gene was observed in *Arabidopsis* [7], and AtAMT1;1, AtAMT1;2, and AtAMT1;3 successfully restored the normal growth of the yeast mutant that is deficient in NH_4_^+^ uptake systems. Subsequent studies of AMT transporters have been identified in diverse plant species, including *Lotus japonicus* (*L*. *japonicus*) [8, 9], *Lycopersicon esculentum* (*L*. *esculentum*) [10–12], *Populus trichocarpa* (*P*. *trichocarpa*) [13], *Oryza sativa* (*O. sativa*) [14, 15], *Triticum aestivum* (*T. aestivum*) [16], *Alternanthera philoxeroides* (*A. philoxeroides*) [17], and so on, which have been characterized in *Xenopus* oocytes or yeast mutant. The AMT family transporters can be divided into two subfamilies: AMT1 and AMT2 [3–5]. In particular, the phosphorylation of amino acid residues of AMT proteins is a recognized means, by which NH_4_^+^ uptake activities were regulated [18, 19]. In *Arabidopsis*, the phosphorylation of the threonine (Thr) residue in the C-terminal tail region of AtAMT1.1 (Thr^460^), AtAMT1.2 (Thr^472^), and AtAMT1.3 (Thr^464^) led to the loss of NH_4_^+^ transport activity in response to increasing external NH_4_^+^ supplies [18–21], providing a novel regulatory mechanism that NH_4_^+^ transport can be rapidly shut-off under high NH_4_^+^ supply conditions.

Functional studies of plant AMT members were mainly focused on the AMT1 family, especially of annual model plants. However, molecular mechanisms towards NH_4_^+^ uptake and transport in fruit trees are largely rare but were just observed in citrus [22] and pear [23, 24]. The biological and physiological functions of AMT2 members remain unknown. Favorably, the knowledge on NH_4_^+^ uptake and transport in model plants provides valuable insights into the investigation of their roles in fruit trees.

As one of the most popular *Rosaceae* fruit crops, peach (*Prunus persica*) has its genome sequenced [25, 26]. In this study, we isolated and characterized a putative AMT2 family gene from peach (entitled as *PpeAMT3;4*) and determined that PpeAMT3;4 is a typical HATS-mediated AMT member responsible for NH_4_^+^ uptake in peach roots. Nonetheless, our findings provide a molecular basis of NH_4_^+^ uptake for fruit trees.

## 2. Materials and Methods

### 2.1. Plant Material and Growth Condition

‘Feicheng' peach seedlings grown in the Key Laboratory of Molecular Module-Based Breeding of High Yield and Abiotic Resistant Plants in Universities of Shandong in Yantai, China, were used throughout this study. Germinated seedlings with similar growth status were transferred into 1/2 Murashige and Skoog (MS) liquid solution ([17, 27], containing approximately 1 mM NH4Cl), cultivated in a climate-controlled growth cabinet that was maintained under 28°C/23°C and 12/12 h light/dark, with 60% relative humidity.

For the NH_4_^+^ deficiency treatments, NH_4_^+^ was omitted from the 1/2 MS medium. For the NH_4_^+^ excess treatments, germinated seedlings were grown in 1/2 MS solution containing 20 mM NH_4_Cl (pH 5.8). Seedlings were exposed to treatment for 72 h before quantitative real-time PCR (qRT-PCR) determination.

### 2.2. Cloning of PpeAMT3; 4 Gene in Peach

Protein sequences of poplar AMT genes [13] were used as query to BLAST the Phytozome Peach Genome Database (http://www.phytozome.net) to identify putative homologues in peach. Information of *PpeAMT* genes were listed in Supplemental Table 1. Coding sequence (CDS) of the *PpeAMT3;4* gene was downloaded from the database, and the first 18 bp sequence from the ATG codon and the last 19 bp sequence from the TAA codon were chosen as the forward primer and reverse primer, respectively (Table 1). Total RNA of ‘Feicheng' peach seedling was extracted using MiniBEST Plant RNA Extraction Kit (TaKaRa, Dalian, China) and reverse transcribed into the 1st cDNA using PrimeScript™ RT reagent Kit (TaKaRa, Dalian, China). Normal gene amplification was carried out to obtain the CDS region of *PpeAMT3;4* and then sequenced in Sanon Biotech (Shanghai, China).

### 2.3. Phylogenetic Analysis of PpeAMT Genes in Peach

Full-length amino acid sequences of PpeAMT transporters were aligned by ClustalX2.1 and imported into the Molecular Evolutionary Genetics Analysis (MEGA) package version 4.1. Phylogenetic analyses were conducted according to the description of Liang et al. [28]. Branch lengths indicate the corresponding phylogenetic distances. Sequence logos of AMT1 and AMT2 subfamilies were shown on the right, generated using WEBLOGO (http://weblogo.berkeley.edu/logo.cgi). The grand average of hydropathicity (GRAVY) and aliphatic index analyses of PpeAMT3;4 were calculated using the ProtParam tool (http://web.expasy.org/protparam/), as described by Liang et al. [28].

### 2.4. qRT-PCR Assays

Specific expression primers for *PpeAMT3;4* and *Ubiquitin* (control gene) were designed using the NCBI/Primer-BLAST online server. Primer sequences were listed in Table 1. According to the description of Liang et al. [28], qRT-PCR analyses were carried out on 7500 Real-Time PCR System (Applied Biosystems, New York, USA), using SYBR Premix Ex Taq reaction kit (TaKaRa, Dalian, China). To calculate RT-qPCR efficiency and the starting template concentration for each sample, the linear regression of the log (fluorescence) per cycle number data was used according to the description of Gazzarrini et al. [29]. The relative expression levels were presented after normalization to the internal control *Ubiquitin* from three independent biological repeats.

### 2.5. Functional Determination of PpeAMT3;4 in Yeast Mutant 31019b

The recombinant plasmid pYES2-*AMT3;4* was constructed by cloning the CDS region of the *PpeAMT3;4* gene into the pYES2 expression vector [17], using the primer pairs (Table 1, *Kpn* I site was introduced and underlined in the forward primer, *Not* I site was introduced and underlined in the reverse primer). The yeast strain 31019b (*MATa mepl△ mep2△::LEU2 mep3△::KanMX2 ura3*, [7, 13, 17]) was transformed with pYES2 harboring the CDS of *PpeAMT3;4*. Yeast complementation tests were carried out as described in previous reports [7, 13, 17]. Yeast strain 31019b was transformed with pYES2 or pYES2-*AMT3;4*, respectively. The growth of yeast cells were determined in the yeast nitrogen base (YNB) liquid medium, supplemented with 0.02, 0.2, or 2 mM NH_4_^+^ as the sole N source (pH 5.8). Pictures were taken at the 3rd day after incubation at 30°C. The growth of transformed yeast cells in the YNB liquid medium was checked by determining absorbance at 600 nm [29].

### 2.6. ^15^N Uptake Kinetics Assay

^15^N labeled NH_4_^+^ uptake assays were carried out according to the description of Guo et al. [17]. Yeast cells transformed with pYES2-MT3;4 or pYES2 were grown in the YNB liquid medium, supplemented with ^15^N labeled NH_4_Cl for 10 min. NH_4_^+^ content was determined by Flash-2000 Delta VADVADTAGE Mass Spectrometer (Thermo Fisher, Waltham, Massachusetts, USA). Michaelis-Menten kinetic constants (*K*_*m*_ and *V*_max_) were calculated as described by Guo et al. [17].

### 2.7. Statistical Analysis

All data were statistically analyzed using independent samples' *t*-test in SPSS 13.0 software (SPSS Chicago, Ilinois, USA). Details are described in figure legends. *Asterisks* indicate statistical differences between plants under control and stress treatment (^∗^*P* < 0.05, ^∗∗^*P* < 0.01).

## 3. Results

### 3.1. Characteristics and Phylogenetic Tree Construction of PpeAMT Transporters in Peach

**Figure 1:**
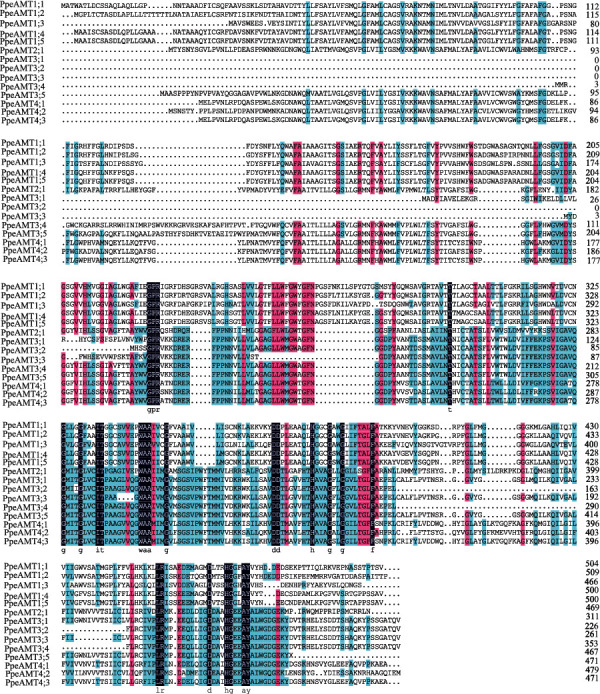
Amino acid sequence alignment of PpeAMT proteins in peach. Multiple sequence alignment of PpeAMT proteins were carried out via ClustalX2.1 software.

**Figure 2:**
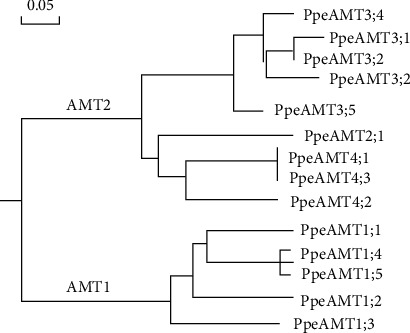
Phylogenetic tree of PpeAMT proteins in peach. A maximum likelihood (ML) tree was constructed by multiple alignment of PpeAMT proteins using ClustalX2.1 and MEGA7.0 software. The tree was based on 1000 bootstrap replicates neighbor-joining method. The PpeAMT family members were divided into two subgroups (AMT1 and AMT2, marked in blue).

By BLAST searching of the Phytozome Peach Genome Database, 14 putative *PpeAMT* genes were screened and identified [27]. Pr7otein domain verification analyses showed that all *PpeAMT* transporters contain the NH_4_^+^ transporter transmembrane domain (PF00909). Information of these *PpeAMT* genes, including gene ID, gene location, CDS (coding sequence) length, and peptide length, are listed in Supplemental Table 1. Notably, the amino acid sequences of PpeAMT proteins shared an overall identity of 51.09% (Figure 1). To confirm the evolutionary relationships of PpeAMT proteins, a maximum likelihood (ML) phylogenetic tree was generated based on the alignment of the amino acid sequences of PpeAMT proteins. All peach AMT transporters were classified into 2 major subgroups: AMT1 and AMT2 (Figure 2), each with 5 and 9 members, respectively. The AMT2 subgroup in peach was further divided into three subclades, including PpeAMT2 (1 member), PpeAMT3 (5 members), and PpeAMT4 (3 members) (Figure 2).

### 3.2. Cloning and Expression Analysis of PpeAMT3;4

As a member of the AMT2 subgroup in peach, the nucleotide sequence of *PpeAMT3;4* CDS was amplified from ‘Feicheng' peach seedlings, using the specific primer pairs and the 1st cDNA template. According to the sequencing results, *PpeAMT3;4* possessed a coding sequence of 1065 bp, encoding a polypeptide of 355 amino acids with the deduced molecular weight of 39.09 kDa. Instability index assay implicated that PpeAMT3;4 was stable and alkalescent, with the theoretical PI value of 8.55. Moreover, both the GRAVY and aliphatic index analysis indicated that PpeAMT3;4 was a hydrophilic protein.

### 3.3. Expression Profiles of PpeAMT3;4

**Figure 3:**
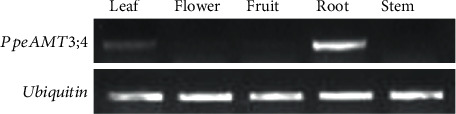
Expression profiles of *PpeAMT3;4* in peach. Reverse-transcribed PCR analysis was carried out using different cDNA templates from tissues of 7-year-old ‘Feicheng' peach trees.

We further performed reverse-transcribed PCR to determine the expression profiles of *PpeAMT3;4* in different tissues of 7-year-old ‘Feicheng' trees. Results showed that *PpeAMT3;4* was majorly expressed in roots, followed by leaves, and hardly observed in stems, flowers, and fruits (Figure 3).

**Figure 4:**
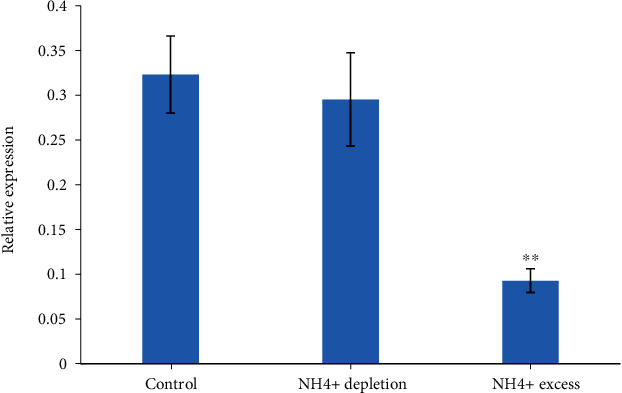
qRT-PCR analysis of *PpeAMT3;4* in roots of 1-month-old ‘Feicheng' seedlings under different NH_4_^+^ supplies. The relative expression level of *PpeAMT3;4* was presented after normalization to the internal control. Data are the means of values obtained from three independent biological repeats ± SE.

To investigate the role of *PpeAMT3;4* in maintaining NH_4_^+^ homeostasis in peach, we analyzed the expression profiles of *PpeAMT3;4* via the qRT-PCR analysis. Results showed that the expression of PpeAMT3;4 was significantly decreased by NH_4_^+^ excess in roots of ‘Feicheng' seedlings but had no response to NH_4_^+^ deficiency (Figure 4).

### 3.4. Functional Determination of PpeAMT1;1 in Yeast Mutant

**Figure 5:**
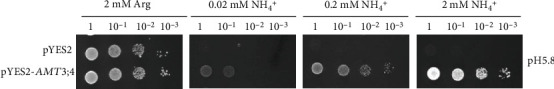
Functional determination of PpeAMT3;4-mediated NH_4_^+^ uptake in yeast. Yeast cells grown on the YNB medium supplemented with 2 mM Arg as the sole N source was used as the positive control. Yeast strains 31019 b transformed with pYES2 or pYES2-*AMT3;4* were grown in the YNB medium, supplemented with different concentrations of NH_4_Cl. Final diluted concentrations are indicated by 1, 10^−1^, 10^−2^, and 10^−3^.

We further carried out functional determination of PpeAMT3;4 via complementation of the yeast mutant 31019b [6, 13, 17, 30]. Results showed that yeast cells harboring pYES2 or pYES2-*PpeAMT3;4* grew well, with similar growth status, on the YNB medium that contains 2 mM Arg as the sole N source (Figure 5). Yeast cells harboring pYES2 could not grow well on the YNB medium containing 0.02, 0.2, or 2 mM NH_4_Cl under pH 5.8, while yeast cells harboring pYES2-*PpeAMT3;4* grew normally on the YNB medium supplemented under 0.2 and 2 mM NH_4_Cl (Figure 5). These findings imply that PpeAMT3;4 is involved in NH_4_^+^ in yeast, which may be responsible for NH_4_^+^ uptake in peach roots.

### 3.5. ^15^N Uptake Kinetics Assay of ApAMT3;4 in Yeast Cells

**Figure 6:**
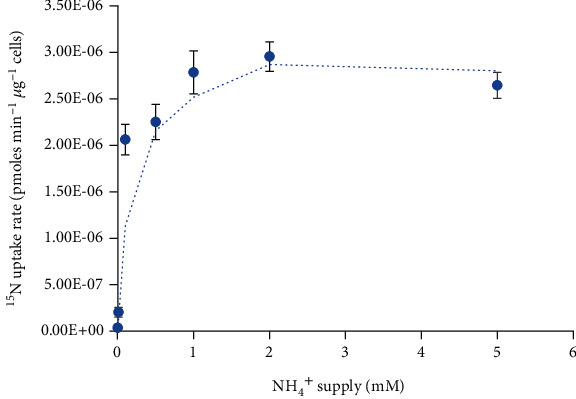
^15^N-labeled NH_4_^+^ uptake analysis of PpeAMT3;4 in yeast. Yeast cells transformed with pYES2 or pYES2-*AMT3;4* were grown in the YNB liquid medium supplemented with different concentrations of ^15^N-labeled NH_4_Cl. Values are presented as mean ± SE, *n* = 3. Error bars within the plot symbols were not visible.

To verify the proposition that PpeAMT3;4 really functions as an active NH_4_^+^ transporter, we determined its NH_4_^+^ uptake activity and the exact amount of ^15^N-labeled NH_4_^+^ uptake in yeast cells via the ^15^N isotope labeling method. Yeast cells were grown in the NYB liquid medium labeled with ^15^N-labeled NH_4_Cl, ranging from 0.02 mM to 2 mM. Affinity constant analysis showed that PpeAMT3;4 exhibited a *K*_*m*_ value of 86.3 *μ*M and a *I*_max_ value of 3.69 *μ*mol min^−1^ *μ*g^−1^, respectively (Figure 6). These findings indicate that PpeAMT3;4 was primarily involved in HATS-mediated NH_4_^+^ uptake in peach roots.

## 4. Discussion

NH_4_^+^ is the preferred form of N transport for lower energetic cost in assimilation [3–5, 29], while excess NH_4_^+^ is toxic to plant growth and development [30, 31]. In higher plants, high-affinity AMT1 subgroup genes encode NH_4_^+^ transporters with a *K*_*m*_ value of micromolar grade involved in the HATS for NH_4_^+^ uptake [12]. However, the molecular mechanism towards the AMT2 subgroup genes is largely rare. In this present study, we initially isolated and determined a high-affinity AMT2 subgroup transporter gene in peach, which helps to investigate the biological role of AMT2 family transporters in fruit trees.

Transcript levels of AMT1 genes are closely related to the N nutrition status of the plant, and NH_4_^+^ starvation mainly induced the expression levels of most *AMT1* genes, may it be a clue of ‘starvation response' for plants to grow under suboptimal N-source conditions [3, 16, 22]. However, *PpeAMT3;4* was mainly expressed in roots and was nonresponsive to NH_4_^+^ starvation in peach roots in this study but reduced significantly by excessive NH_4_^+^ stress (Figures 3 and 4). These findings implies that PpeAMT3;4 may be an active NH_4_^+^ transporter at normal NH_4_^+^ supplies, even at extremely low NH_4_^+^ conditions, which is an indispensable AMT2 transporter responsible for NH_4_^+^ uptake in peach roots. If the NH_4_^+^ supply reached an excessive state, PpeAMT3;4 began to stop its NH_4_^+^ uptake and transport capacity that are sufficient enough for internal N metabolism and usage.

Heterologous expression studies using NH_4_^+^ uptake defective yeast mutant or *Xenopus* oocytes indicate that AMT1 family genes encode high-affinity transporters [3, 4, 15], which play key roles in high-affinity NH_4_^+^ uptake from soils via the roots. While molecular mechanisms or biological functions of AMT2 subgroup members are extremely rare, in this present study, PpeAMT3;4 could utilize the external NH_4_^+^ in yeast cells (Figure 5), and ^15^N labeled NH_4_^+^ uptake kinetics analysis exhibited a *K*_*m*_ value of 86.3 *μ*M (Figure 6). Nonetheless, PpeAMT3;4 is a functional AMT2 transporter responsible for NH_4_^+^ uptake in peach roots, especially under normal and low external NH_4_^+^ conditions.

## 5. Conclusions

In this study, we isolated and characterized *PpeAMT3;4*, an AMT2 family gene from peach, and determined its function in yeast mutant. The *PpeAMT3;4* gene was majorly expressed in peach roots, whose expression was decreased under NH_4_^+^ excess but had no response to NH_4_^+^ deficiency. Functional determination and ^15^N-labeled NH_4_^+^ uptake kinetics assay in yeast cells indicate that ApAMT3;4 was a typical high-affinity transporter, with a *K*_*m*_ value of 86.3 *μ*M and a *I*_max_ value of 3.69 *μ*mol min^−1^ *μ*g^−1^, which can uptake external NH_4_^+^ in yeast cells. Nonetheless, PpeAMT3;4 might be a NH_4_^+^ transporter involved in NH_4_^+^ uptake in peach roots. This study not only provides a technological system to uncover the biological function of AMT2 transporters in fruit trees but also reveals molecular basis for NH_4_^+^ uptake and N nutrition mechanisms.

## Figures and Tables

**Table 1:** Primer sequences used in this work.

Purpose	Primer (5′-3′)	Amplicon (bp)
Amplication of *PpeAMT3;4* CDS	F: ATGATGCGAGGTTGGTGC R: TTAGACCACCTGAGTGGCA	1065
Specific expression primers of *PpeAMT3;4*	F: TGTCGGAGCTTTCAGCTTGT R: TGAAACCTGCCCATCCCATC	224
Specific expression primers of *Ubiquitin*	F: AGGCTAAGATCCAAGACAAAGA R: CCACGAAGACGAAGCACTAAG	145
Construction of pYES2-*AMT3;4* plasmid	F: GAGCGGTACCATGATGCGAGGTTGGTGC R: GCGAGCGGCCGCTTAGACCACCTGAGTGGC	1065

## Data Availability

The data used to support the findings of this study are included within the article.

## Supplementary Materials

Supplementary Materials Supplemental Table 1: information of *PpeAMT* family genes in peach.
