# Supplementary material for: Cloning and Functional Determination of Ammonium Transporter PpeAMT3;4 in Peach
Source: Biomed Res Int. 2020 Dec 3;2020:2147367. doi: 10.1155/2020/2147367 (PMC7732375; doi:10.1155/2020/2147367)
Supplement: Supplementary Materials — Supplemental Table 1: information of PpeAMT family genes in peach. [file 2147367.f1.doc]

Supplemental Table 1. Information of *PpeAMT* family genes in peach

| Gene | Locus name | Gene location | Scaffold  distribution | ORF (bp) | Amino acids |
| --- | --- | --- | --- | --- | --- |
| *PpeAMT1;1* | ppa004542m | 3655374-3657084 | 1 | 1515 | 504 |
| *PpeAMT1;2* | ppa004450m | 28182013-28183551 | 2 | 1530 | 509 |
| *PpeAMT1;3* | ppa005341m | 176876-178278 | 6 | 1401 | 466 |
| *PpeAMT1;4* | ppa004613m | 18874579-18876081 | 8 | 1503 | 500 |
| *PpeAMT1;5* | ppa015730m | 43101574-43103076 | 1 | 1503 | 500 |
| *PpeAMT2;1* | ppa022420m | 17889600-17891484 | 7 | 1410 | 469 |
| *PpeAMT3;1* | ppa008980m | 28342145-28345100 | 4 | 939 | 312 |
| *PpeAMT3;2* | ppa020093m | 28437353-28439602 | 4 | 684 | 227 |
| *PpeAMT3;3* | ppa019792m | 28462529-28465742 | 4 | 789 | 262 |
| *PpeAMT3;4* | ppa022674m | 28279751-28284237 | 4 | 1065 | 354 |
| *PpeAMT3;5* | ppa004749m | 28131401-28135102 | 4 | 1482 | 467 |
| *PpeAMT4;1* | ppa004845m | 24446309-24449047 | 6 | 1470 | 471 |
| *PpeAMT4;2* | ppa027111m | 38371072-38372953 | 1 | 1443 | 480 |
| *PpeAMT4;3* | ppa005235m | 19984263-19986069 | 8 | 1416 | 471 |

Note: Gene names, locus names, gene location, scaffold disrtibution, ORF and amino acids numbers were collected from the online database of Phytozome Peach Genome Database (http://WWW.phytozome.net).
